# A commentary on “Clinical efficacy of different surgical approaches in the treatment of thoracolumbar tuberculosis: a multicenter retrospective case–control study with a minimum 10-year follow-up”

**DOI:** 10.1097/JS9.0000000000003003

**Published:** 2025-07-08

**Authors:** Yonggang Lu, Qiang Zhang

**Affiliations:** a Department of Spine Ward of Orthopedics and Traumatology, Qiannan Traditional Chinese Medicine Hospital, Guizhou, China

*Dear Editor,*

We read with great interest the recent article titled “Clinical efficacy of different surgical approaches in the treatment of thoracolumbar tuberculosis: a multicenter retrospective case–control study with a minimum 10-year follow-up”^[1]^ published in the *International Journal of Surgery*. This study aims to assess the long-term clinical effectiveness of three distinct surgical techniques for the treatment of thoracolumbar TB. The findings indicated that in cases with isolated lesions and lesser angular abnormalities, an anterior-only approach is advised. A posterior-only or anterior–posterior combined approach is recommended for individuals with multisegmental lesions and bigger angular deformities; the posterior-only technique is preferred. Future studies must, however, address a number of important concerns.

First, the study offers a thorough explanation of the steps involved in three surgical techniques; however, it does not standardize the surgical methods, including the choice of bone graft materials (autograft or allograft) and the kind of internal fixation system (such as PEEK or titanium alloy)^[2]^. These factors can influence the surgical outcomes and the incidence of complications. Additionally, the study does not mention the use of intraoperative navigation or robotic-assisted techniques, which could further enhance surgical precision and safety. Future research is recommended to clarify technical details and explore the application of new technologies in spinal tuberculosis surgery.

Second, while the study recorded the occurrence of problems (12.73% in the AO group, 16.98% in the PO group, and 22.06% in the AP group), it did not examine the connection between complications and baseline patient characteristics like diabetes or osteoporosis or surgical procedures^[3]^. For instance, the higher complication rate in the AP group might be linked to greater surgical trauma, but the impact of factors like incision length and surgical duration on complications was not quantified. Additionally, the long-term effects of early (e.g., infection) and late (e.g., internal fixation failure) complications were not distinguished. Future studies should use multivariate regression analysis to identify the risk factors for complications.

Third, the study did not incorporate objective measures of motor function (e.g., gait analysis) or patient-reported quality of life scales (e.g., SF-36), even though it employed VAS, ODI, and ASIA scores to evaluate functional recovery. Moreover, there was no statistically significant difference in the rates of neurological recovery (84.62% in the AO group, 87.10% in the PO group, and 83.72% in the AP group), but specific improvements in neurological functions (such as sensation or movement) were not analyzed. Future studies are recommended to integrate multimodal assessment tools to comprehensively reflect the patient’s functional recovery.

This study system compared the long-term efficacy of three surgical approaches for treating spinal tuberculosis, but it had shortcomings in terms of study design, technical details, complication analysis, functional assessment, long-term follow-up, and economic analysis. Future surgical strategies need to be further optimized through RCT design, standardized techniques, multimodal evaluation, and health economics research. All of this was noted in relation to the TITAN guideline, which calls for transparency in the reporting of artificial intelligence^[4]^.

## Data Availability

No data was used in this Letter to the Editor.

## Research registration unique identifying number (UIN)

Name of the registry: not applicable. Unique identifying number or registration ID: not applicable. Hyperlink to your specific registration (must be publicly accessible and will be checked): not applicable.
